# Supplementary figures and images for: Alterations in children’s sub-dominant gut microbiota by HIV infection and anti-retroviral therapy
Source: PLoS One. 2021 Oct 11;16(10):e0258226. doi: 10.1371/journal.pone.0258226 (PMC8504761; doi:10.1371/journal.pone.0258226)

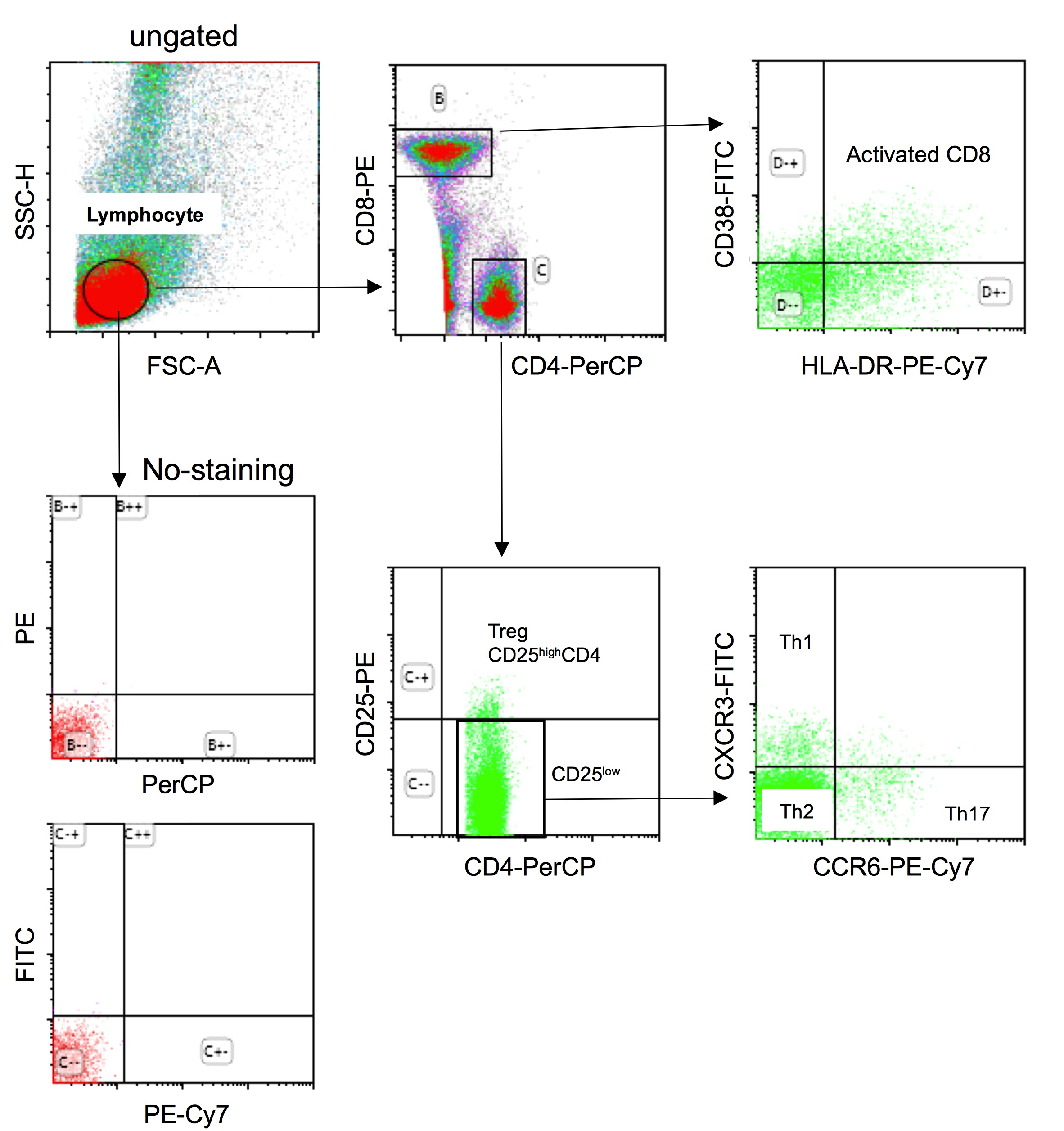

Supplement: S1 Fig — CD8+ cell activations was defined as the CD38+HLA-DR+ population. Regulatory T (Treg) cells were defined as CD25highCD4+ cells, Th1 as CXCR3+CCR6−CD25lowCD4+ cells, Th2 as CXCR3−CCR6−CD25lowCD4+ cells, and Th17 as CXCR3−CCR6+CD25lowCD4+ cells. Unstained cells were used for gating controls. (TIF) [file pone.0258226.s001.tif]
