# Supplementary material for: Alterations in children’s sub-dominant gut microbiota by HIV infection and anti-retroviral therapy
Source: PLoS One. 2021 Oct 11;16(10):e0258226. doi: 10.1371/journal.pone.0258226 (PMC8504761; doi:10.1371/journal.pone.0258226)
Supplement: S1 Table — Values are the median counts (IQR), based on RT-qPCR, expressed in units of log10 cells/g feces. The Lactobacillus spp. counts were obtained with RT-qPCR and are expressed as the sum of the six subgroups and two species; ART: anti-retroviral therapy; C.: Clostridium; L.: Lactobacillus; B.: Bacteroides. P-values in bold are statistically significant, based on the Man-Whitney U test. (DOCX) [file pone.0258226.s002.docx]

**S1 Table. Number of bacteria in fecal samples from each study group.**

|  |  |  |  |  | *P* values | | |
| --- | --- | --- | --- | --- | --- | --- | --- |
| **Target bacteria** | HIV(−)  n = 20 | HIV(+)  n = 30 | ART(+)  n = 29 |  | HIV(+) vs.  HIV(−) | ART(+) vs.  HIV(−) | HIV(+) vs.  ART(+) |
| **Total** | 10.5 (10.2−10.8) | 10.6 (10.3−10.7) | 10.6 (10.3−10.7) |  | 0.81 | 0.98 | 0.81 |
| **Phylum Firmicutes** | 10.3 (9.6−10.5) | 10.2 (9.9−10.4) | 10.1 (9.7−10.3) |  | 0.69 | 0.13 | **0.02** |
| *C. coccoides* group | 9.7 (9.1−10.0) | 9.6 (9.4−9.9) | 9.4 (9.0−9.8) |  | 0.86 | 0.28 | 0.052 |
| *C. leptum* subgroup | 10.1 (9.5−10.3) | 9.9 (9.5−10.2) | 9.7 (9.2−10.0) |  | 0.81 | 0.06 | 0.07 |
| *C. difficile* | 1.15 | 1.15 | 1.15 |  | 0.35 | 0.35 | **0.046** |
| *C. perfringens* | 6.9 (5.5−7.6) | 5.8 (4.0−6.7) | 5.8 (3.8−7.0) |  | **0.02** | **0.048** | 1.00 |
| *Lactobacillus* spp. | 7.2 (6.0−7.9) | 8.0 (6.6−8.9) | 8.4 (7.4−9.1) |  | **0.02** | **0.001** | 0.23 |
| *L. casei*subgroup | 2.3 (1.5-5.1) | 4.9 (4.4-5.6) | 4.8 (3.1-5.2) |  | **0.01** | **0.08** | 0.33 |
| *L. gasseri*subgroup | 5.1 (2.5-6.2) | 6.3 (5.4-7.2) | 6.4 (5.9-6.6) |  | **0.01** | **0.01** | 0.81 |
| *L. plantarum* subgroup | 6.1 (3.1-7.1) | 5.6 (4.4-7.0) | 6.4 (4.4-7.2) |  | 0.80 | 0.50 | 0.33 |
| *L. reuteri* subgroup | 5.1 (3.5-6.4) | 6.3 (5.0-7.0) | 6.4 (5.5-7.1) |  | **0.02** | **0.01** | 0.62 |
| *L. ruminis* subgroup | 4.0 (1.2-6.4) | 7.7 (4.7-8.6) | 8.4 (6.2-9.1) |  | **0.002** | **< 0.001** | 0.18 |
| *L. sakei*subgroup | 1.2 (1.2-4.4) | 1.2 (1.2-3.1) | 4.9 (1.2-5.7) |  | 0.90 | **0.05** | **0.01** |
| *L. brevis* | 1.2 (1.2-3.2) | 1.2 (1.2-4.0) | 1.2 |  | 0.65 | **0.01** | **0.004** |
| *L. fermentum* | 5.4 (2.0-6.6) | 6.3 (5.1-7.3) | 5.5 (2.0-6.1) |  | **0.047** | 0.72 | **0.004** |
| *Streptococcus* | 8.7 (8.0−9.0) | 8.9 (8.5−9.3) | 8.5 (8.2−9.0) |  | 0.23 | 0.82 | 0.10 |
| *Enterococcus* | 6.9 (6.2−7.6) | 7.5 (6.7−8.0) | 5.3 (1.4−7.2) |  | 0.14 | **0.01** | **<0.001** |
| *Staphylococcus* | 4.3 (3.7−5.3) | 4.1 (3.4−4.6) | 4.1 (3.5−4.9) |  | 0.14 | 0.34 | 0.68 |
| **Phylum Actinobacteria** | 9.9 (9.4−10.1) | 9.7 (9.3−9.9) | 10.2 (9.9−10.4) |  | 0.10 | **0.01** | **<0.001** |
| *Bifidobacterium* | 9.7 (9.1−10.0) | 9.4 (8.8−9.8) | 10.1 (9.7−10.3) |  | 0.14 | **0.03** | **<0.001** |
| *Atopobium* cluster | 9.3 (8.9−9.6) | 9.0 (8.5−9.3) | 9.7 (9.5−9.8) |  | **0.048** | **0.002** | **<0.001** |
| **Phylum Bacteroidetes** | 9.7 (9.2−10.2) | 9.6 (9.1−10.1) | 9.7 (9.2−10.0) |  | 0.51 | 0.60 | 0.96 |
| *B. fragilis* group | 9.1 (8.4−9.5) | 9.0 (8.5−9.4) | 8.8 (8.3−9.1) |  | 0.77 | **0.03** | **0.04** |
| *Prevotella* | 9.1 (4.0−10.0) | 8.2 (2.45−10.1) | 9.6 (8.6−10.0) |  | 0.58 | 0.48 | 0.06 |
| **Phylum Proteobacteria** | 8.1 (7.8−8.4) | 8.1 (7.7−8.5) | 7.5 (6.7−8.1) |  | 0.72 | **0.003** | **0.002** |
| *Enterobacteriacea* | 8.1 (7.8−8.4) | 8.1 (7.7−8.5) | 7.5 (6.7−8.1) |  | 0.72 | **0.003** | **0.002** |
| *Pseudomonas* | 1.45 | 1.45 | 1.45 |  | 0.38 | 0.83 | 0.47 |
