# Supplementary material for: Alterations in children’s sub-dominant gut microbiota by HIV infection and anti-retroviral therapy
Source: PLoS One. 2021 Oct 11;16(10):e0258226. doi: 10.1371/journal.pone.0258226 (PMC8504761; doi:10.1371/journal.pone.0258226)
Supplement: S2 Table — Values are the detection frequency, defined as the % of samples that harbored detectable microbiota, among all samples in a given group. The Lactobacillus spp. counts were obtained with RT-qPCR and are expressed as the sum of the six subgroups and two species; ART: anti-retroviral therapy; C.: Clostridium; L.: Lactobacillus; B.: Bacteroides; P-values in bold are statistically significant, based on the Chi-square test or Fisher’s exact probability test. (DOCX) [file pone.0258226.s003.docx]

**S2 Table. Detection frequency of bacteria in fecal samples from each study group.**

|  |  |  |  | *P* value | | |
| --- | --- | --- | --- | --- | --- | --- |
| **Target bacteria** | HIV(−)  n = 20 | HIV(+)  n = 30 | ART(+)  n = 29 | HIV(+) vs.  HIV(−) | ART(+) vs.  HIV(−) | HIV(+) vs.  ART(+) |
| **Total** | 100 | 100 | 100 |  |  |  |
| **Phylum Firmicutes** |  |  |  |  |  |  |
| *C. coccoides* group | 90.0 | 96.7 | 93.1 | 0.56 | 1.00 | 0.61 |
| *C. leptum* subgroup | 100 | 100 | 100 |  |  |  |
| *C. difficile* | 10.0 | 20.0 | 3.4 | 0.45 | 0.56 | 0.10 |
| *C. perfringens* | 100 | 96.7 | 86.2 | 1.00 | 0.14 | 0.20 |
| *Lactobacillus* spp. | 100 | 100 | 100 |  |  |  |
| *L. casei*subgroup | 50.0 | 90.0 | 79.3 | **0.003** | 0.06 | 0.30 |
| *L. gasseri*subgroup | 85.0 | 90.0 | 96.6 | 0.67 | 0.29 | 0.61 |
| *L. plantarum* subgroup | 80.0 | 90.0 | 100 | 0.42 | **0.02** | 0.24 |
| *L. reuteri* subgroup | 90.0 | 96.7 | 100 | 0.56 | 0.16 | 1.00 |
| *L. ruminis* subgroup | 60.0 | 86.7 | 100 | **0.04** | **<0.001** | 0.11 |
| *L. sakei*subgroup | 30.0 | 30.0 | 55.2 | 1.00 | 0.14 | 0.07 |
| *L. brevis* | 45.0 | 46.7 | 17.2 | 1.00 | 0.05 | **0.03** |
| *L. fermentum* | 65.0 | 83.3 | 69.0 | 0.18 | 1.00 | 0.23 |
| *Streptococcus* | 90.0 | 100 | 86.2 | 0.16 | 1.00 | 0.052 |
| *Enterococcus* | 95.0 | 86.7 | 51.7 | 0.64 | **0.001** | **0.005** |
| *Staphylococcus* | 100 | 86.7 | 93.1 | 0.14 | 0.51 | 0.67 |
| **Phylum Actinobacteria** |  |  |  |  |  |  |
| *Bifidobacterium* | 100 | 100 | 100 |  |  |  |
| *Atopobium* cluster | 100 | 96.7 | 100 | 1.00 |  | 1.00 |
| **Phylum Bacteroidetes** |  |  |  |  |  |  |
| *B. fragilis* group | 100 | 100 | 100 |  |  |  |
| *Prevotella* | 75.0 | 70 | 100 | 0.76 | **0.01** | **0.002** |
| **Phylum Proteobacteria** |  |  |  |  |  |  |
| *Enterobacteriaceae* | 100 | 100 | 96.6 |  |  |  |
| *Pseudomonas* | 5.0 | 13.3 | 6.9 |  |  |  |
