# Supplementary material for: Alterations in children’s sub-dominant gut microbiota by HIV infection and anti-retroviral therapy
Source: PLoS One. 2021 Oct 11;16(10):e0258226. doi: 10.1371/journal.pone.0258226 (PMC8504761; doi:10.1371/journal.pone.0258226)
Supplement: S3 Table — ART: anti-retroviral therapy; Treg: regulatory T cell; C.: Clostridium; B.: Bacteroides; P-values in bold are statistically significant, based on Spearman’s rank correlation analysis. (DOCX) [file pone.0258226.s004.docx]

**S3 Table. Association between gut mictobiota and age, ART duration, immune status, and use of cotrimoxazole in children.**

| Bacteria |  | Age (years) | | | ART  Duration  (years) | CD4^+^ cell  counts | | | Th1  counts | | | Th2  counts | | | Th17  counts | | | Treg  counts | | | CD8^+^ cell  counts | | | Activated  CD8^+^ cells % | | | Use of cotrimoxazole | |
| --- | --- | --- | --- | --- | --- | --- | --- | --- | --- | --- | --- | --- | --- | --- | --- | --- | --- | --- | --- | --- | --- | --- | --- | --- | --- | --- | --- | --- |
|  |  | HIV(−) | HIV(+) | ART(+) | ART(+) | HIV(−) | HIV(+) | ART(+) | HIV(−) | HIV(+) | ART(+) | HIV(−) | HIV(+) | ART(+) | HIV(−) | HIV(+) | ART(+) | HIV(−) | HIV(+) | ART(+) | HIV(−) | HIV(+) | ART(+) | HIV(−) | HIV(+) | ART(+) | HIV(+) | ART(+) |
| Total | Rho | -0.21 | -0.11 | -0.01 | 0.06 | 0.12 | 0.36 | 0.06 | -0.09 | 0.40 | 0.13 | 0.24 | 0.35 | 0.10 | -0.19 | 0.44 | -0.08 | -0.03 | 0.36 | 0.14 | -0.31 | 0.29 | -0.11 | -0.08 | -0.26 | 0.13 | -0.13 | -0.06 |
|  | *P* | 0.37 | 0.55 | 0.97 | 0.74 | 0.61 | 0.05 | 0.77 | 0.70 | **0.03** | 0.51 | 0.31 | 0.06 | 0.62 | 0.44 | **0.02** | 0.69 | 0.89 | 0.05 | 0.47 | 0.18 | 0.12 | 0.57 | 0.74 | 0.17 | 0.51 | 0.50 | 0.75 |
| *C. coccoides* group | Rho | -0.08 | -0.31 | -0.18 | -0.10 | 0.14 | 0.51 | 0.21 | -0.14 | 0.54 | 0.29 | 0.23 | 0.50 | 0.21 | -0.15 | 0.54 | 0.09 | -0.10 | 0.49 | 0.45 | -0.18 | 0.36 | 0.07 | -0.19 | -0.26 | 0.02 | 0.06 | 0.14 |
|  | *P* | 0.75 | 0.09 | 0.34 | 0.61 | 0.56 | **0.004** | 0.27 | 0.55 | **0.002** | 0.12 | 0.34 | **0.005** | 0.28 | 0.53 | **0.002** | 0.64 | 0.68 | **0.006** | **0.01** | 0.46 | 0.05 | 0.73 | 0.41 | 0.17 | 0.92 | 0.76 | 0.48 |
| *C. leptum* subgroup | Rho | -0.17 | -0.13 | -0.07 | 0.10 | -0.01 | 0.34 | 0.20 | -0.11 | 0.36 | 0.21 | 0.09 | 0.34 | 0.21 | -0.28 | 0.41 | 0.10 | -0.13 | 0.36 | 0.32 | -0.34 | 0.23 | 0.06 | -0.008 | -0.32 | -0.05 | 0.04 | 0.18 |
|  | *P* | 0.48 | 0.50 | 0.72 | 0.63 | 0.96 | 0.07 | 0.29 | 0.65 | 0.05 | 0.27 | 0.72 | 0.06 | 0.27 | 0.23 | **0.03** | 0.62 | 0.60 | 0.05 | 0.09 | 0.14 | 0.23 | 0.76 | 0.98 | 0.09 | 0.81 | 0.86 | 0.36 |
| *C. difficile* | Rho | -0.10 | -0.01 | 0.16 | -0.05 | 0.14 | -0.08 | -0.18 | 0.27 | 0.11 | -0.25 | 0.06 | -0.12 | -0.16 | 0.33 | -0.06 | -0.23 | 0.17 | -0.16 | -0.02 | 0.40 | 0.22 | -0.27 | 0.34 | 0.38 | -0.20 | 0.12 | -0.13 |
|  | *P* | 0.69 | 0.97 | 0.41 | 0.82 | 0.56 | 0.67 | 0.35 | 0.24 | 0.57 | 0.19 | 0.82 | 0.52 | 0.41 | 0.16 | 0.74 | 0.24 | 0.49 | 0.40 | 0.91 | 0.08 | 0.25 | 0.16 | 0.14 | **0.04** | 0.29 | 0.52 | 0.51 |
| *C. perfringens* | Rho | 0.23 | 0.27 | 0.39 | 0.75 | -0.21 | -0.06 | 0.39 | -0.15 | 0.05 | 0.09 | -0.22 | -0.02 | 0.40 | -0.07 | -0.003 | 0.42 | -0.17 | 0.14 | 0.20 | 0.17 | -0.19 | -0.22 | 0.12 | -0.30 | -0.49 | -0.30 | -0.47 |
|  | *P* | 0.33 | 0.15 | **0.03** | **<0.001** | 0.38 | 0.75 | **0.04** | 0.53 | 0.80 | 0.66 | 0.36 | 0.94 | **0.03** | 0.76 | 0.99 | **0.03** | 0.48 | 0.47 | 0.29 | 0.48 | 0.32 | 0.24 | 0.61 | 0.11 | **0.01** | 0.11 | **0.01** |
| *Lactobacillus* spp. | Rho | 0.12 | 0.14 | 0.20 | -0.04 | -0.31 | -0.26 | -0.07 | -0.12 | -0.08 | 0.15 | -0.37 | -0.28 | -0.11 | -0.24 | -0.08 | 0.005 | -0.15 | -0.05 | -0.08 | 0.28 | -0.26 | 0.17 | 0.04 | 0.06 | 0.31 | -0.20 | -0.06 |
|  | *P* | 0.62 | 0.47 | 0.31 | 0.86 | 0.19 | 0.16 | 0.74 | 0.61 | 0.68 | 0.44 | 0.11 | 0.13 | 0.57 | 0.30 | 0.70 | 0.98 | 0.53 | 0.78 | 0.67 | 0.23 | 0.17 | 0.39 | 0.87 | 0.75 | 0.10 | 0.30 | 0.75 |
| *Streptococcus* | Rho | 0.59 | -0.03 | -0.26 | 0.21 | -0.04 | 0.01 | 0.14 | -0.30 | -0.004 | 0.06 | -0.005 | 0.05 | 0.17 | -0.05 | 0.001 | 0.09 | -0.13 | -0.03 | 0.14 | -0.15 | 0.05 | -0.07 | -0.41 | -0.07 | 0.003 | -0.25 | -0.08 |
|  | *P* | **0.006** | 0.86 | 0.17 | 0.27 | 0.86 | 0.94 | 0.46 | 0.20 | 0.98 | 0.75 | 0.98 | 0.79 | 0.39 | 0.83 | 1.00 | 0.65 | 0.60 | 0.88 | 0.48 | 0.53 | 0.81 | 0.71 | 0.08 | 0.71 | 0.99 | 0.18 | 0.68 |
| *Enterococcus* | Rho | 0.61 | 0.19 | -0.16 | -0.05 | 0.20 | 0.04 | 0.02 | 0.24 | -0.19 | -0.09 | 0.11 | 0.06 | 0.05 | 0.42 | -0.32 | -0.02 | 0.19 | -0.13 | -0.04 | 0.13 | 0.17 | -0.07 | -0.06 | 0.06 | -0.23 | -0.16 | 0.12 |
|  | *P* | **0.005** | 0.31 | 0.42 | 0.81 | 0.41 | 0.83 | 0.92 | 0.30 | 0.32 | 0.66 | 0.66 | 0.77 | 0.80 | 0.06 | 0.08 | 0.9 | 0.42 | 0.48 | 0.85 | 0.59 | 0.36 | 0.72 | 0.79 | 0.77 | 0.23 | 0.40 | 0.53 |
| *Staphylococcus* | Rho | -0.07 | -0.47 | -0.08 | 0.42 | -0.10 | 0.18 | 0.46 | 0.12 | -0.001 | 0.44 | -0.16 | 0.21 | 0.37 | -0.09 | 0.015 | 0.58 | 0.006 | -0.07 | 0.45 | 0.02 | 0.17 | 0.33 | 0.2- | 0.24 | -0.39 | 0.01 | -0.13 |
|  | *P* | 0.78 | **0.009** | 0.68 | **0.02** | 0.67 | 0.34 | **0.01** | 0.61 | 0.96 | **0.02** | 0.51 | 0.26 | **0.047** | 0.71 | 0.94 | **0.001** | 0.98 | 0.71 | **0.02** | 0.94 | 0.37 | 0.08 | 0.41 | 0.20 | **0.04** | 0.97 | 0.52 |
| *Bifidobacterium* | Rho | -0.31 | 0.13 | 0.12 | 0.10 | -0.03 | 0.007 | 0.08 | -0.09 | 0.05 | 0.10 | 0.02 | -0.01 | 0.10 | -0.16 | 0.14 | -0.11 | -0.10 | 0.03 | -0.02 | -0.01 | -0.04 | 0.005 | 0.11 | 0.09 | 0.03 | -0.03 | -0.13 |
|  | *P* | 0.18 | 0.49 | 0.53 | 0.60 | 0.91 | 0.97 | 0.68 | 0.71 | 0.81 | 0.62 | 0.95 | 0.96 | 0.61 | 0.49 | 0.46 | 0.56 | 0.67 | 0.90 | 0.93 | 0.97 | 0.82 | 0.98 | 0.65 | 0.64 | 0.90 | 0.89 | 0.49 |
| *Atopobium* cluste*r* | Rho | -0.20 | 0.10 | -0.08 | -0.21 | -0.46 | -0.04 | -0.16 | -0.33 | -0.06 | 0.02 | -0.41 | -0.05 | -0.14 | -0.51 | 0.27 | -0.24 | -0.33 | 0.34 | -0.03 | -0.39 | -0.10 | 0.17 | 0.21 | 0.03 | 0.44 | 0.03 | 0.05 |
|  | *P* | 0.40 | 0.60 | 0.66 | 0.29 | **0.04** | 0.84 | 0.42 | 0.15 | 0.74 | 0.94 | 0.07 | 0.80 | 0.46 | **0.02** | 0.15 | 0.20 | 0.15 | 0.07 | 0.88 | 0.09 | 0.61 | 0.39 | 0.38 | 0.87 | **0.02** | 0.89 | 0.82 |
| *B. fragilis* group | Rho | -0.24 | 0.13 | 0.11 | 0.08 | -0.05 | 0.24 | 0.36 | 0.05 | 0.21 | 0.23 | 0.02 | 0.27 | 0.34 | -0.16 | 0.23 | 0.27 | 0.03 | 0.18 | 0.40 | -0.04 | 0.33 | 0.36 | 0.33 | -0.08 | -0.04 | 0.03 | 0.10 |
|  | *P* | 0.31 | 0.49 | 0.56 | 0.69 | 0.84 | 0.20 | 0.06 | 0.84 | 0.26 | 0.23 | 0.93 | 0.15 | 0.07 | 0.51 | 0.22 | 0.16 | 0.92 | 0.33 | **0.03** | 0.87 | 0.06 | 0.05 | 0.16 | 0.67 | 0.83 | 0.89 | 0.61 |
| *Prevotella* | Rho | -0.14 | -0.20 | 0.08 | 0.35 | 0.26 | 0.04 | 0.17 | -0.04 | 0.15 | 0.08 | 0.35 | 0.01 | 0.24 | -0.1 | 0.15 | 0.11 | 0.07 | 0.21 | 0.19 | -0.26 | 0.18 | -0.41 | -0.39 | -0.16 | -0.08 | 0.07 | -0.28 |
|  | *P* | 0.55 | 0.30 | 0.69 | 0.07 | 0.26 | 0.82 | 0.37 | 0.88 | 0.42 | 0.68 | 0.13 | 0.96 | 0.21 | 0.68 | 0.43 | 0.58 | 0.76 | 0.26 | 0.33 | 0.26 | 0.34 | **0.03** | 0.09 | 0.38 | 0.67 | 0.70 | 0.15 |
| *Enterobacteriaceae* | Rho | 0.57 | 0.17 | -0.01 | 0.12 | 0.05 | -0.06 | -0.004 | 0.07 | -0.17 | 0.04 | -0.06 | -0.03 | -0.04 | 0.31 | -0.24 | 0.05 | 0.07 | 0.08 | 0.02 | -0.01 | -0.18 | -0.05 | -0.21 | -0.03 | -0.12 | -0.24 | 0.28 |
|  | *P* | **0.008** | 0.38 | 0.96 | 0.53 | 0.84 | 0.75 | 0.98 | 0.77 | 0.38 | 0.85 | 0.81 | 0.88 | 0.84 | 0.19 | 0.21 | 0.81 | 0.79 | 0.66 | 0.92 | 0.97 | 0.35 | 0.81 | 0.37 | 0.87 | 0.54 | 0.20 | 0.15 |
| *Pseudomonas* | Rho | -0.30 | -0.26 | 0.003 | -0.22 | 0.06 | 0.50 | -0.28 | -0.10 | 0.30 | -0.10 | 0.06 | 0.52 | -0.29 | 0.10 | 0.19 | -0.21 | 0.18 | 0.09 | -0.04 | 0.02 | 0.21 | 0.25 | 0.06 | -0.21 | 0.20 | 0.19 | 0.41 |
|  | *P* | 0.20 | 0.16 | 0.99 | 0.26 | 0.80 | **0.005** | 0.15 | 0.68 | 0.11 | 0.59 | 0.81 | **0.004** | 0.13 | 0.68 | 0.32 | 0.28 | 0.45 | 0.64 | 0.84 | 0.93 | 0.26 | 0.18 | 0.80 | 0.27 | 0.30 | 0.32 | **0.03** |
