# Supplementary material for: Alterations in children’s sub-dominant gut microbiota by HIV infection and anti-retroviral therapy
Source: PLoS One. 2021 Oct 11;16(10):e0258226. doi: 10.1371/journal.pone.0258226 (PMC8504761; doi:10.1371/journal.pone.0258226)
Supplement: S4 Table — Values are the median counts (IQR), based on RT-qPCR, expressed in units of log10 cells/g feces; ART: anti-retroviral therapy; C.: Clostridium; B.: Bacteroides; P-values in bold are statistically significant, based on the Mann−Whitney U test. (DOCX) [file pone.0258226.s005.docx]

**S4 Table. Number of bacteria in fecal samples from the HIV(+) and ART(+) groups stratified by the use of cotrimoxazole.**

|  |  | HIV(+) | |  |  | ART(+) | |
| --- | --- | --- | --- | --- | --- | --- | --- |
| **Target bacteria** | Use (n=15) | No use (n=15) | *P* value |  | Use (n=9) | No use (n=20) | *P* value |
| **Total** | 10.6 (10.3−10.7) | 10.5 (10.3−10.8) | 0.49 |  | 10.5 (10.3−10.7) | 10.6 (10.4−10.8) | 0.74 |
| **Phylum Firmicutes** | 10.2 (10.0−10.4) | 10.2 (9.9−10.6) | 0.72 |  | 10.1 (9.7−10.3) | 10.0 (9.5−10.3) | 0.64 |
| *C. coccoides* group | 10.7 (9.5−9.9) | 9.6 (9.4−9.9) | 0.76 |  | 9.5 (9.2−9.8) | 9.4 (8.9−9.8) | 0.47 |
| *C. leptum* subgroup | 10.0 (9.5−10.2) | 9.8 (9.3−10.3) | 0.85 |  | 9.7 (9.5−10.0) | 9.7 (9.1−10.0) | 0.35 |
| *C. difficile* | 1.15 | 1.15 | 0.51 |  | 1.15 | 1.15 | 0.50 |
| *C. perfringens* | 5.3 (3.7−6.3) | 6.1 (5.0−6.8) | 0.11 |  | 3.4 (2.2−5.4) | 6.2 (4.8−7.5) | **0.01** |
| *Lactobacillus* spp. | 7.8 (6.6−8.6) | 8.2 (6.6−9.0) | 0.29 |  | 8.4 (7.6−8.9) | 8.5 (7.2−9.4) | 0.74 |
| *Streptococcus* | 8.6 (8.4−9.1) | 9.0 (8.5−9.4) | 0.18 |  | 8.6 (4.7−9.2) | 8.5 (8.2−9.0) | 0.67 |
| *Enterococcus* | 7.2 (6.7−7.9) | 7.6 (6.6−8.2) | 0.40 |  | 5.3 (1.4−7.5) | 3.7 (1.4−7.1) | 0.52 |
| *Staphylococcus* | 4.3 (3.1−4.9) | 4.0 (3.7−4.3) | 0.97 |  | 4.0 (3.2−4.7) | 4.2 (3.6−4.9) | 0.51 |
| **Phylum Actinobacteria** | 9.7 (9.5−10.0) | 9.5 (9.3−9.9) | 0.72 |  | 10.2 (9.9−10.3) | 10.2 (10.0−10.5) | 0.67 |
| *Bifidobacterium* | 9.6 (8.5−9.9) | 9.2 (9.0−9.7) | 0.89 |  | 10.1 (9.6−10.1) | 10.0 (9.7−10.3) | 0.48 |
| *Atopobium* cluster | 8.9 (8.5−9.4) | 9.1 (8.4−9.3) | 0.89 |  | 9.7 (9.5−9.7) | 9.6 (9.4−9.8) | 0.81 |
| **Phylum Bacteroidetes** | 9.6 (9.1−10.1) | 9.6 (8.8−10.1) | 0.49 |  | 9.3 (8.8−9.7) | 9.8 (9.3−10.0) | 0.14 |
| *B. fragilis* group | 9.1 (8.5−9.5) | 8.9 (8.4−9.3) | 0.89 |  | 8.8 (8.3−9.1) | 8.8 (8.2−9.0) | 0.60 |
| *Prevotella* | 9.2 (2.5−10.1) | 6.4 (2.5−10.1) | 0.69 |  | 8.8 (8.3−9.7) | 9.7 (8.8−10.0) | 0.14 |
| **Phylum Proteobacteria** | 8.0 (7.4−8.4) | 8.3 (8.0−8.6) | 0.19 |  | 8.0 (7.4−8.3) | 7.2 (6.7−7.9) | 0.14 |
| *Enterobacteriacea* | 8.0 (7.4−8.4) | 8.3 (8.0−8.6) | 0.19 |  | 8.0 (7.4−8.3) | 7.2 (6.7−7.9) | 0.14 |
| *Pseudomonas* | 1.45 | 1.45 | 0.31 |  | 1.45 (1.45−2.8) | 1.45 (1.45−1.45) | 0.03 |
