# Supplementary material for: Alterations in children’s sub-dominant gut microbiota by HIV infection and anti-retroviral therapy
Source: PLoS One. 2021 Oct 11;16(10):e0258226. doi: 10.1371/journal.pone.0258226 (PMC8504761; doi:10.1371/journal.pone.0258226)
Supplement: S5 Table — ART: anti-retroviral therapy; Treg: regulatory T cells; Beta: regression coefficient; SE: standard error. Stepwise multiple linear regression analysis was not done, since no factor with P < 0.05 was found in the simple linear regression analysis. (DOCX) [file pone.0258226.s006.docx]

**S5 Table. Linear regression analysis of *Clostridium coccoides* group with immune status in the HIV(+) group.**

|  | **Unadjusted linear regression** | | | **Adjusted linear regression** | | |
| --- | --- | --- | --- | --- | --- | --- |
| **Variable** | **Beta** | **SE** | ***P*-value** | **Beta** | **SE** | ***P*-value** |
| Th1 count | 0.223 | 0.004 | 0.237 |  |  |  |
| Th2 count | 0.245 | 0.001 | 0.193 |  |  |  |
| Th17 count | 0.233 | 0.010 | 0.215 |  |  |  |
| Treg count | 0.149 | 0.010 | 0.434 |  |  |  |

ART: anti-retroviral therapy; Treg: regulatory T cells; Beta: regression coefficient; SE: standard error.

Stepwise multiple linear regression analysis was not done, since no factor with *P* < 0.05 was found in the simple linear regression analysis.
